# Carcinome épidermoide du pénis

**DOI:** 10.11604/pamj.2016.23.91.8487

**Published:** 2016-03-15

**Authors:** Asmaa Naim, Fatim-Zahra Zakouri

**Affiliations:** 1 Centre Régional d'Oncologie, Alhouceima, Maroc

**Keywords:** Carcinome épidermoide, pénis, chimiothérapie, Epidermoid carcinoma, penis, chemotherapy

## Abstract

Penile tumors are the rarest male urogenital tract tumors (1%). Squamous cell carcinoma is the most common histologic type (95%). Radical surgical excision, though often mutilating, remains the best treatment for localized stages, in terms of local control (6% local recurrence). There are other therapeutic weapons and their possible indications should be adapted to the locoregional extension and at a distance from the primary tumor. We report the case of Mr AS aged 61, without particular pathological antecedents, who consulted, 6 years before, for an ulcerated burgeoning in the anterior surface of the penile bleeding on contact, which extended along the penis, without involving the glans penis. Evolution was marked by tumor progression with extension to the anterior pelvic wall. The patient went into a critical state of hemorrhagic shock. After stabilizing the hemodynamic status, a biopsy of the penis lesion confirmed the diagnosis of well-differentiated squamous cell carcinoma. An evaluation of the extent of loco-regional recurrence and distant metastasis showed pulmonary micronodules in the right lung. The case was discussed at the Multidisciplinary Consultative Meeting and patient was deemed inoperable and offered palliative chemotherapy. The prognosis of advanced stages of penis cancer is reserved; their frequency in our context is related to the modesty of patients, which explains the causes of delay in medical examination. Only socio-cultural awareness allows early diagnosis and, therefore, a better prognosis.

## Image en médecine

Les tumeurs du pénis sont les tumeurs les plus rares de l'appareil génito-urinaire masculin (1%). Le carcinome épidermoide représente le type histologique le plus fréquent (95%). La chirurgie radicale d'exérèse, quoique souvent mutilante, reste le meilleur traitement des stades localisés en termes de contrôle local (6% de récidive locale). Il existe d'autres armes thérapeutiques dont les indications sont à adapter à l'extension locorégionale et à distance de la Tumeur primitive. Nous rapportons l'observation de Mr A.S âgé de 61 ans sans antécédents pathologiques particuliers qui a présenté 6 ans auparavant, une lésion ulcéro- bourgeonnante au niveau de la face antérieure de la verge saignante au contact qui s'est étendue à l'ensemble de la verge épargnant le gland (A), l'évolution a été marquée par la progression tumorale avec extension à la paroi pelvienne antérieure (B). Le patient a été admis dans un tableau de choc hémorragique, après stabilisation de son état hémodynamique, une biopsie de la lésion de la verge a confirmé le carcinome épidermoide bien différencié. Le bilan d'extension a objectivé des micronodules pulmonaires droits. Le cas a été discuté en Réunion de Concertation Pluridisciplinaire est le patient a été jugé inopérable d'où une chimiothérapie palliative a été retenue. Le pronostic des stades avancés des cancers de la verge est réservé, leur fréquence dans notre contexte est en rapport avec la pudeur des patients ce qui explique les délais de consultations. Seule une sensibilisation socioculturelle permettrait un diagnostic précoce et donc un meilleur pronostic.

**Figure 1:**
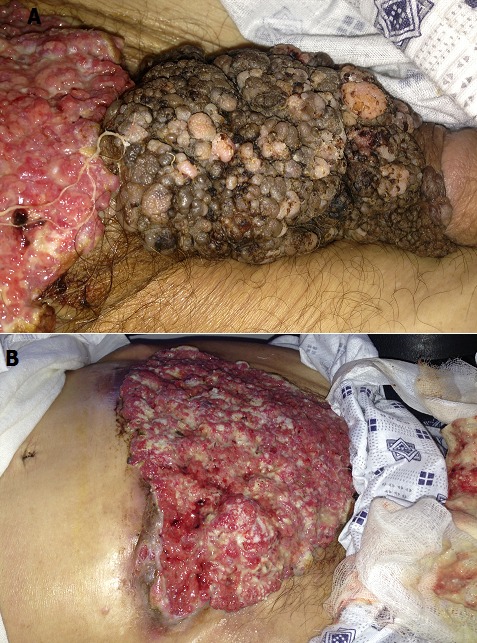
(A) tumeur ulcéro-bourgeonnante du pénis épargnant le gland; (B) tumeur du pénis étendue sur la paroi abdominale antérieure sous ombilicale

